# EGF Potentiation of VEGF Production Is Cell Density Dependent in H292 EGFR Wild Type NSCLC Cell Line

**DOI:** 10.3390/ijms151017686

**Published:** 2014-09-30

**Authors:** Daniel J. Ranayhossaini, Jin Lu, John Mabus, Alexis Gervais, Russell B. Lingham, Natalie Fursov

**Affiliations:** 1 Biologics Research, Janssen Research and Development, Welsh & McKean Roads, Spring House, PA 19477, USA; E-Mails: djr61@pitt.edu (D.J.R.); jlu9@its.jnj.com (J.L.); agervais@its.jnj.com (A.G.); rlingham@its.jnj.com (R.B.L.); 2 Department of Pharmacology & Chemical Biology, University of Pittsburgh, 200 Lothrop Street, Pittsburgh, PA 15213, USA; 3 Cardiovascular & Metabolic Disease, Janssen Research and Development, Welsh & McKean Roads, Spring House, PA 19477, USA; E-Mail: jmabus@its.jnj.com

**Keywords:** non-small cell lung cancer, vascular endothelial growth factor, epidermal growth factor receptor, cell density

## Abstract

Non-small cell lung cancer (NSCLC) affects millions of patients each year worldwide. Existing therapies include epidermal growth factor receptor (EGFR) inhibition using small molecules or antibodies with good efficacy. Unfortunately, intrinsic and acquired resistance to EGFR therapy remains a persistent complication for disease treatment. A greater understanding of the role of EGFR in NSCLC etiology is crucial to improving patient outcomes. In this study, the role of EGFR in tumor angiogenesis was examined in H292 NSCLC cells under the pretense that confluent cells would exhibit a more angiogenic and growth-centered phenotype. Indeed, confluent H292 cells potentiated endothelial cell angiogenesis in co-culture models in an EGFR-dependent manner. While confluent H292 cells did not exhibit any change in EGFR protein expression, EGFR localization to the extracellular membrane was increased. EGFR membrane localization coincided with a comparable potentiation of maximal EGFR phosphorylation and was followed by a 3-fold increase in vascular endothelial growth factor A (VEGF-A) production as compared to subconfluent cells. EGFR-mediated VEGF-A production was determined to be dependent on signal transducer and activator of transcription 3 (STAT3) activation and not phosphoinositide 3-kinase (PI3K) signaling. These results identify unique cell density dependent phenotypes within a monoclonal NSCLC cell line and provide a potential mechanism of resistance to anti-EGFR therapy in metastatic NSCLC.

## 1. Introduction

Non-small cell lung cancer (NSCLC) is the most common type of lung cancer and has an unacceptably poor prognosis and represents 18% of all cancer deaths [1]. A large subset of NSCLC patients exhibit enhanced signaling of the epidermal growth factor receptor (EGFR) and/or the hepatocyte growth factor receptor (cMet), with EGFR inhibition being an effective clinical treatment option for NSCLC [2,3]. Nevertheless, resistance to EGFR-targeted therapies remains a constant struggle in NSCLC treatment, demanding an improved understanding of EGFR’s role in NSCLC cell physiology [1]. Resistance to EGFR targeted therapies is in part a result of mutations in EGFR signaling pathways. Among the many possible mutations, some of the more common occur within EGFR itself, as L858R and T790M, or downstream of EGFR in the Kirsten rat sarcoma viral oncogene homolog (KRAS). These mutations can activate EGFR, promote resistance to EGFR inhibitors (L858R), potentiate ATP affinity (T790M), or constitutively activate EGFR downstream signaling in a ligand independent manner (KRAS mutations) [4,5,6]. While these mutations are critically important in a subset of patients, most NSCLC patients (60%–80%) do not exhibit either EGFR or KRAS mutations and are classified as EGFR wild type [7]. Considering these data, we chose to investigate EGFR regulation in NSCLC line H292 as H292 is devoid of any EGFR or KRAS mutations and as such, is a suitable representative of wild type patients representing the majority of NSCLC cases.

EGFR and cMet are tyrosine kinase receptors initially existing as monomeric transmembrane proteins [8,9]. Among transmembrane proteins, EGFR and cMet are continually recycled through the extracellular and intracellular membranes as a result of such processes as endocytic uptake, concentration in clathrin coated pits, and extracellular membrane targeting [10]. Binding of epidermal growth factor (EGF) or hepatocyte growth factor (HGF), the cognate ligands for EGFR and cMet, respectively, promotes dimerization and phosphorylation of each receptor thereby activating intracellular signaling transduction pathways that include PI3K and STAT3 [8,9,11]. Phosphorylation of the lipid phosphatidylinositol 4,5-bisphosphate PIP2 by PI3K or transcriptional activation by STAT3 both promote cell growth and angiogenic signaling, in part through VEGF production [11,12].

VEGF is an extremely potent secreted angiogenic protein highly specific for endothelial cells that promotes vascular permeability with multiple homologues, including VEGF-A, VEGF-C, and VEGF-D [13,14]. VEGF-C and VEGF-D each share ~30% sequence identity with VEGF-A and promote lymphangiogeneis and lung development whereas VEGF-A serves to promote angiogenesis and vascular permeability [15]. VEGF secretion by tumors is a critical signaling event that provides a blood supply for the growing tumor by mobilizing endothelial cells in the peripheral vasculature [16]. Indeed, without VEGF secretion, tumors are unable to grow beyond a few millimeters in diameter [17]. We hypothesized that NSCLC cells cultured at high density would recognize the necessity for angiogenesis to mediate further cell expansion and produce VEGF at a greater rate than cells cultured at a low density. Numerous signaling pathways could be responsible for this process, with EGFR and cMet-mediated pathways being likely candidates in NSCLC.

While the role of EGFR and cMet as important targets promoting tumor growth is well understood, little is known about relationship between EGFR and cMet signaling and cell density. Previous investigations in healthy cells have established that EGFR degradation contributes to contact-inhibition of cell growth [18]. However, the loss of contact-inhibition is a hallmark of malignant tumor phenotypes and it is likely that the role of EGFR is markedly different in malignant *vs.* benign cells [19]. Furthermore, it is established that contact-inhibition is acutely dependent on EGF levels and that elevated EGF enables cells to override contact-inhibition [20]. These observations indicate that EGF sensitive tumor cell lines, such as those prevalent in NSCLC, may demonstrate an enhanced ability to override contact inhibition through EGFR signaling, thus perpetuating tumor growth beyond normal physical constraints.

Early tumors are localized, cohesive cell aggregates with their nutritional requirements fulfilled by interstitial fluid. As tumors exceed the nutritional capabilities of interstitial fluid, the tumor begins two processes necessary for its continued growth survival: Invasion into its surroundings and angiogenesis. We hypothesized that these distinctly different process mandate that phenotypically identical, monoclonal NSCLC cells (cell line H292) adapt to their different functions and phenotypically separate. Furthermore, as both EGFR and cMet are major oncogenic proteins in NSCLC with major contributions to tumor angiogenesis and contact-inhibition, we focused our efforts on determining whether EGFR and/or cMet mechanistically support phenotypic distinctions in monoclonal tumor cells.

The work presented here identifies a novel synergistic interaction between cell-to-cell contact and EGF signaling as quantified by VEGF-A secretion and angiogenic activity. This process is not a result of increased EGFR expression, but rather an optimization of EGFR organization at the plasma membrane, thus enhancing EGFR phosphorylation and subsequent STAT3 signal transduction and VEGF-A secretion.

## 2. Results and Discussion

### 2.1. Dense Cell Spots Promote Angiogenesis to a Greater Degree than Sparse Cell Lawns

**Figure 1:**
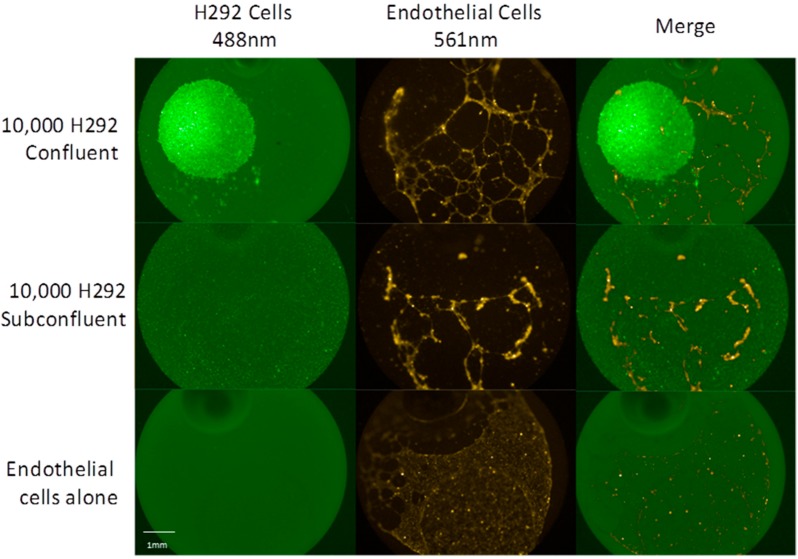
Potentiation of endothelial cell tube formation and angiogenesis in a co-culture model of H292 cells and human microvascular pulmonary endothelial cells (HMPEC). Top, endothelial cells seeded on matrix conditioned by a single spot of 10,000 H292 cells; Middle, endothelial cells seeded on matrix conditioned by subconfluent lawn of 10,000 H292 cells; and Bottom, endothelial cells seeded on matrix devoid of H292 cells.

Little work has been done to investigate phenotypic changes within a previously homogenous population of cells. In an effort to distinguish these phenotypic changes, two novel cell culture models of tumor microenvironments mimicking the dense core of the tumor and the scattered periphery of invading cells were developed. H292, lung epidermoid non-small cell carcinoma, cells were seeded as either a confluent cell spot or a subconfluent cell lawn. In both culture conditions, 10,000 H292 cells were seeded, albeit in very different cell densities. The tumor cells were used to condition a Matrigel matrix for 16 h, after which time human microvascular pulmonary endothelial cells (HMPEC) were seeded on top of the matrix and cultured for 12 h while HMPEC tubulogenesis was monitored using fluorescence microscopy. After 12 h, HMPEC cultured with dense spots of H292 cells exhibited markedly increased tubulogenesis as compared to those cultured with sparse H292 cells (Figure 1).

### 2.2. EGFR Plasma Membrane Localization Is Enhanced in Confluent H292 Cells

**Figure 2:**
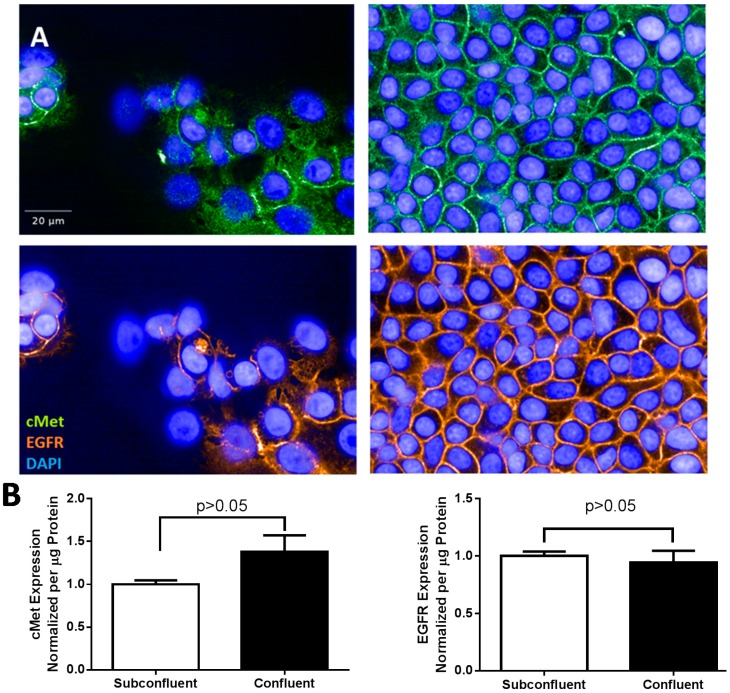
(**A**) Apparent increase in hepatocyte growth factor receptor (cMet) and epidermal growth factor receptor (EGFR) expression in H292 cells seeded at confluence compared to subconfluent H292 cells. Green = cMet, Orange = EGFR, Blue = Hoechst; and (**B**) Unaltered cMet (**left**) and EGFR (**right**) whole cell protein expression as measured by Meso Scale Discovery kit (MSD).

**Figure 3:**
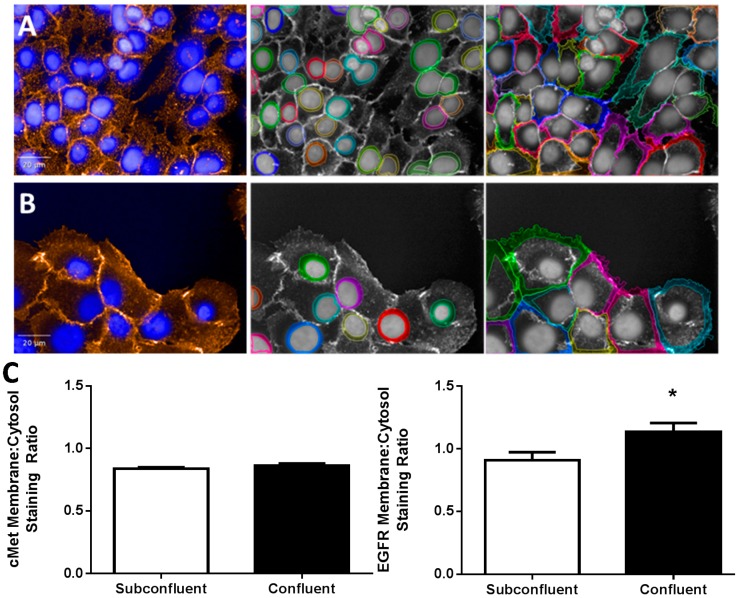
EGFR compartmentalization in subconfluent *vs*. confluent cells. Far left, Orange = EGFR; Blue = Hoechst. (**A**) Separation of cytosolic (**center**) *vs*. extracellular membrane (**right**) staining of EGFR in confluent H292 cells; (**B**) Separation of cytosolic (**center**) *vs*. extracellular membrane (**right**) staining of EGFR in subconfluent H292 cells, various colors on center and right images indicate different objects/cells and (**C**) Membrane, cytosol staining ratio for cMet (**left**) and EGFR (**right**) in subconfluent and confluent H292 cells. * *p* < 0.05, Student’s *t*-test, *n* > 100 cells.

To further understand the different phenotypes of confluent and subconfluent H292 cells, we examined expression of EGFR and cMet, two tyrosine kinases with large bodies of evidence supporting their oncogenicity and ability to potentiate angiogenesis. Imaging data of confluent H292 cells consistently seemed to indicate a greater intensity of EGFR and cMet as compared to subconfluent cells, yet whole cell lysates showed no difference in protein expression levels (Figure 2). Using Harmony image analysis software (Perkin Elmer, Waltham, MA, USA), the nucleus, cytoplasm, and extracellular membrane were separately identified and fluorescence intensity independently quantified from confocal images of stained cells. Independent extracellular membrane *vs.* cytosol fluorescence quantification indicated that EGFR compartmentalization in the extracellular membrane is enhanced in confluent cells (Figure 3). Indeed, previous reports have identified EGFR compartmentalization to the extracellular membrane at places of cell-cell contact in transfected COS and primary A431 cells [21,22]. Interestingly, EGFR compartmentalization occurs at cell junctions between EGFR transfected and EGFR non-transfected cells, indicating that this effect is not merely a result of dual fluorescence from overlapping membranes [21]. These data indicate that either endocytic uptake or extracellular membrane targeting of EGFR, but not cMet, is altered in confluent cells.

### 2.3. EGFR Phosphorylation Is Enhanced in Confluent Cells

**Figure 4:**
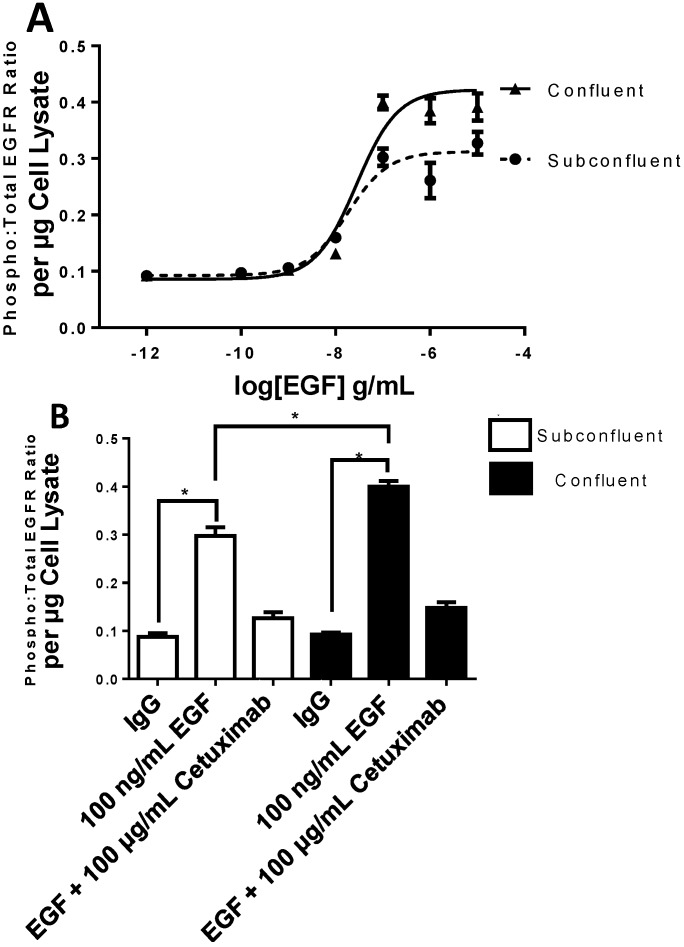
EGFR phosphorylation is enhanced in confluent H292 cells as compared to subconfluent cells. (**A**) Dose dependent increase in EGFR phosphorylation 30 min post epidermal growth factor (EGF) treatment; (**B**) EGFR phosphorylation 30 min post EGF treatment is significantly greater in confluent H292 cells and is prevented by pre-incubation with Cetuximab (EGFR inhibitor). * *p* < 0.05 Student’s *t*-test, *n* = 9.

While EGFR cell junction compartmentalization has been observed previously [21,22], no previous study has investigated the relevance of EGFR localization to cell-cell junctions. We did so by adding increasing concentrations of EGF to H292 cells grown at subconfluence or confluence and determined the ratio of phospho:total EGFR normalized to total cell protein 30 min post EGF ligand treatment. Interestingly, when accounting for total cell protein content, confluent cells exhibited a significant 30% increase in the amount of phosphorylated EGFR at saturation with no change in half maximal effective concentration (EC_50_) as compared to subconfluent cells (Figure 4). EGFR phosphorylation was inhibitable with Cetuximab (EGFR inhibitor) pretreatment, verifying the specificity of this process for EGF treatment. When these data are considered in the context of EGFR extracellular membrane compartmentalization in confluent cells (Figure 3), it is evident that the magnitude of increased membrane compartmentalization in confluent cells mirrors that of EGFR phosphorylation.

### 2.4. Vascular Endothelial Growth Factor (VEGF) A Transcription and Secretion Is Stimulated by Epidermal Growth Factor (EGF) Treatment in Confluent Cells

**Figure 5:**
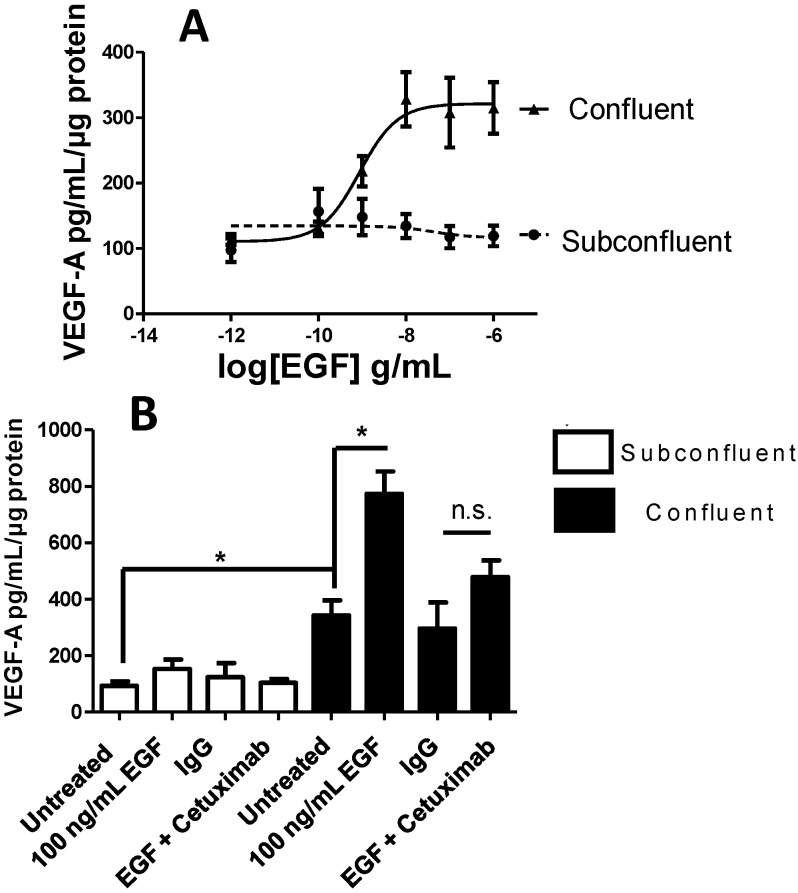
VEGF-A production in confluent *vs*. subconfluent cells treated with EGF. (**A**) Confluent cells exhibit dose dependent increase in VEGF-A production in response to EGF whereas subconfluent cell VEGF-A production was insensitive to EGF treatment; and (**B**) Basal VEGF-A production in confluent cells is greater than that in subconfluent cells and is significantly stimulated by EGF while 30 min pre-incubation with Cetuximab prevented EGF stimulated VEGF-A production. * *p* < 0.05 Student’s *t*-test, n.s. = not significant, *n* = 9.

The initial observation that densely cultured H292 cells supported HPMEC angiogenesis to a greater degree than sparsely cultured H292 cells suggested that angiogenic cytokine secretion is altered between the two cell phenotypes. After 24 h treatment with vehicle, 100 ng/mL EGF and/or 100 μg/mL Cetuximab there was minimal production of VEGF-C, VEGF-D, sFLT1, PIGF and bFGF secretion by H292 cells (data not shown). However, untreated confluent cells produced significantly more VEGF-A as compared to subconfluent cells (342 *vs.* 94 pg/mL/μg protein). No VEGF-A was detected in culture media not exposed to H292 cells (data not shown). Furthermore, when subconfluent cells were treated with EGF no significant change in VEGF-A secretion was detected. In stark contrast, EGF treatment of confluent cells caused a 2-fold increase in VEGF-A secretion (774 *vs.* 342 pg/mL/μg protein, Figure 5). Under both basal and EGF-stimulated conditions, Cetuximab inhibited VEGF-A secretion by H292 cells.

### 2.5. Angiogenesis Promoted by Dense H292 Cell Spots Is EGFR Dependent

**Figure 6:**
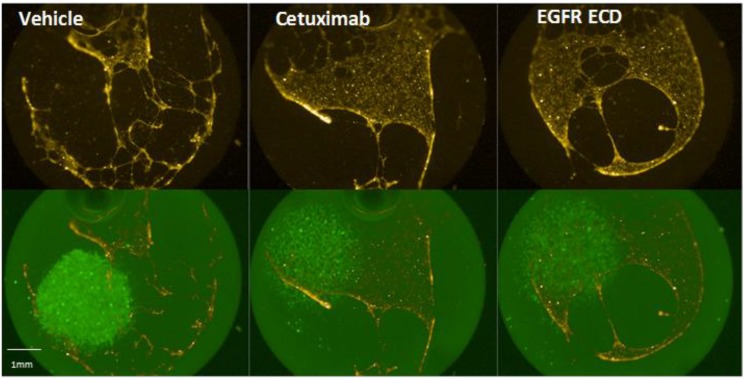
Paracrine effect of H292 cells on HMPEC angiogenesis is EGFR-dependent. Inclusion of either Cetuximab or EGFR Extracellular Domain (ECD, EGF ligand scavenger) in growth matrix prevented paracrine effect of H292 cell spots on HMPEC angiogenesis.

As VEGF production in H292 cells was shown to depend largely on EGFR signaling, we returned to our co-culture model of tumor angiogenesis. Even within the “growth factor reduced” matrix used for our studies, residual EGF can be as high as 0.1 ng/mL, with the potential for significant EGFR activation during extended cell cultures. To account for residual EGF in the matrix, Cetuximab and recombinant EGFR extracellular domain (ECD) were added to the matrix to either inhibit EGFR (Cetuximab) or scavenge remaining EGF in the matrix (EGFR ECD). H292 cells were again seeded as either dense spots or sparse lawns and co-cultured endothelial cell tube formation was monitored. When added to H292 cells prior to Matrigel matrix coating, both EGFR specific inhibitor Cetuximab and recombinant EGFR ECD inhibited endothelial cell tube formation, indicating that confluent H292 cell stimulation of HMPEC angiogenesis is EGFR dependent (Figure 6). Taken in context with the observed VEGF-A stimulation, it is likely that EGFR’s role in HMPEC angiogenesis is through paracrine VEGF-A signaling.

### 2.6. STAT3, but not PI3K, Mediates EGFR-Stimulated VEGF-A Secretion

**Figure 7:**
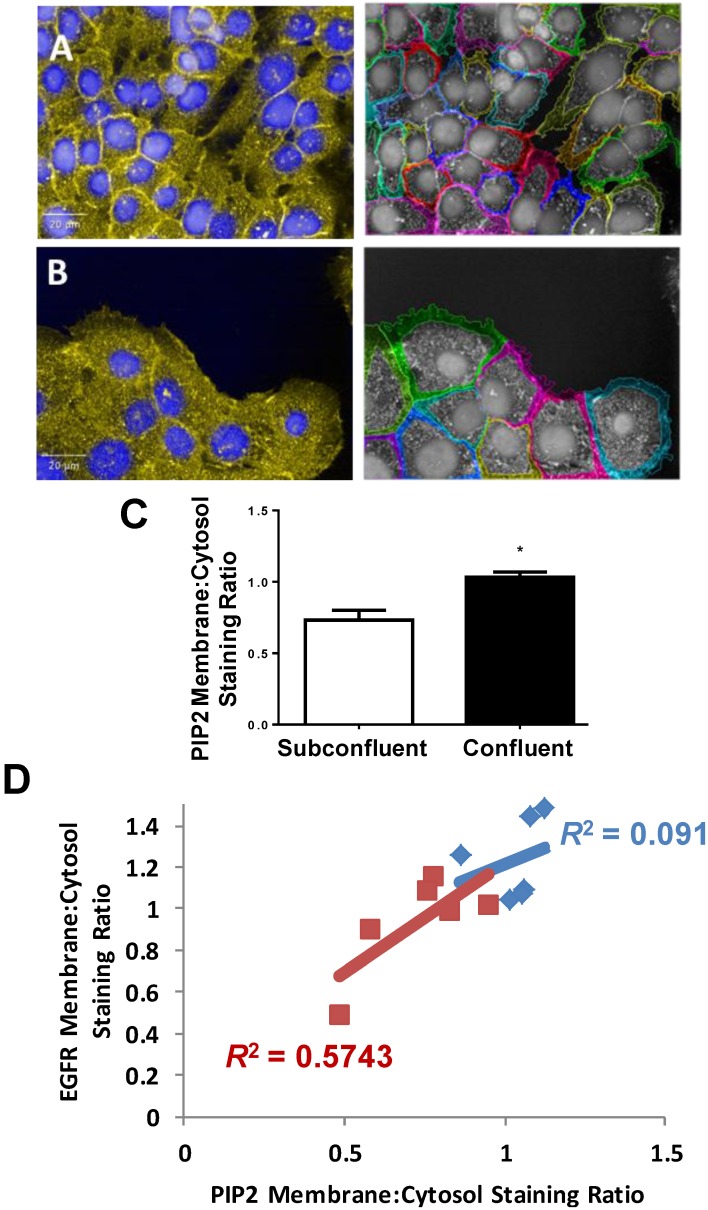
PIP2 compartmentalization in confluent *vs*. subconfluent H292 cells. PIP2 was stained in confluent (**A**) and subconfluent (**B**) H292 cells and membrane compartmentalization was quantified. Images at left Yellow = PIP2, Blue = Hoechst, various colors on right images indicate different objects/cells; (**C**) Image quantification indicated significantly increased PIP2 extracellular membrane compartmentalization; * *p* < 0.05, *n* > 100 cells (**D**) EGFR membrane:cytoplasm staining ratio was graphed against PIP2 membrane:cytoplasm staining ratio per each well. In subconfluent cells, EGFR and PIP2 staining ratios correlated well (red squares) yet not in confluent cells (blue diamonds).

**Figure 8:**
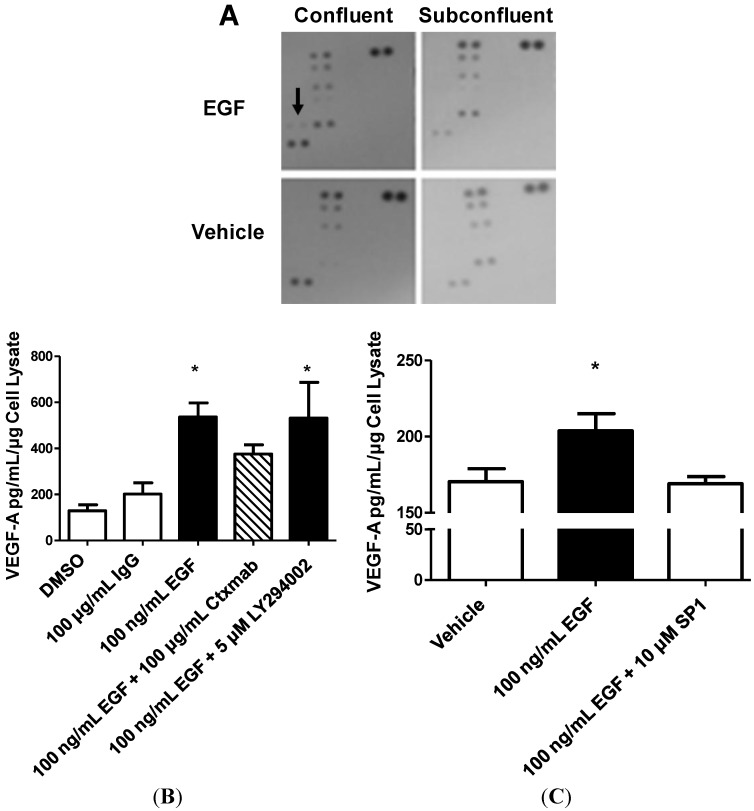
STAT3, but not PI3K, contributes to EGFR-mediated VEGF-A production in confluent cells. (**A**) R&D Systems phospho-protein dot-blot of membranes incubated with equal amounts of cell lysate prepared 30 min after 100 ng/mL EGF treatment on cells seeded as confluent or subconfluent. Arrow indicates STAT3-ser727 phosphorylation; (**B**) VEGF-A production in confluent cells 24 h after indicated treatment, Ctxmab = Cetuximab, * *p* < 0.05, one-way ANOVA with Bonferonni post test; and (**C**) VEGF-A production in confluent cells 24 h after indicated treatment. * *p* < 0.05, students *t*-test *vs*. SP1.

The observed 30% increase in EGFR phosphorylation, while significant, was only a fraction of the 300% increase in VEGF-A production resulting from EGF treatment in confluent cells. To reconcile the surprisingly large gap between enhanced EGFR phosphorylation and VEGF-A production, downstream targets of EGFR with known angiogenic functionality (PI3K and STAT3) were investigated as potential synergistic contributors to EGFR-mediated VEGF-A secretion. We first evaluated the intracellular compartmentalization of PI3K’s substrate, PIP2 and found increased extracellular membrane localization of PIP2 in confluent cells. However, when PIP2 compartmentalization was compared to EGFR compartmentalization, no correlation was observed between PIP2 and EGFR compartmentalization, indicating that PI3K may not play a greater role for EGFR signal transduction in confluent *vs.* subconfluent cells (Figure 7). To test this, confluent H292 cells were treated with PI3K inhibitor LY294002 followed by EGF. LY294002 had no effect on EGF stimulated VEGF-A production, indicating PI3K is not responsible for EGF stimulation of VEGF-A production (Figure 8).

**Figure 9:**
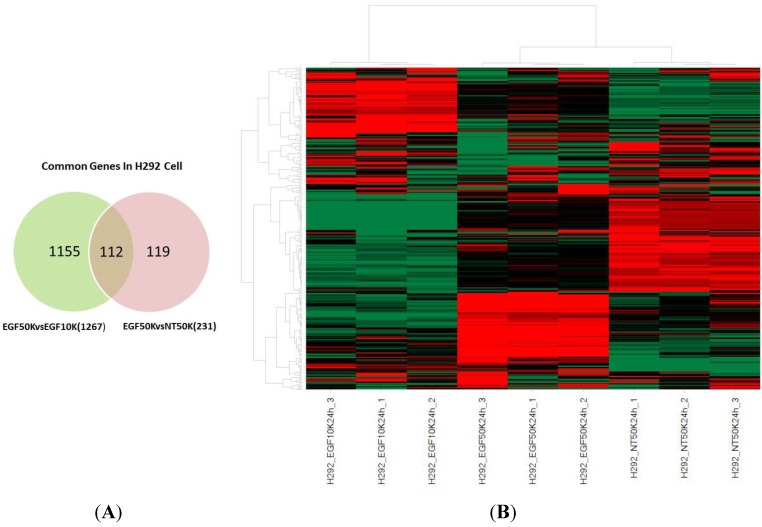
EGF-responsive genes in confluent conditions. (**A**) Venn diagram of gene expression data for EGF-treated confluent (EGF50K), EGF-treated subconfluent (EGF10K), and untreated confluent (NT50K) samples; and (**B**) Heap map of differentially expressed common 112 genes for the samples listed in Table 1. Numbers 1, 2, or 3 at the end of a sample name indicate one of three biologic replicates for that group.

To evaluate STAT3 activation as a result of EGF treatment in confluent cells in conjunction with other potentially relevant proteins, we used phospho-specific proteomic profilers provided by R&D Systems. These profiles consistently indicated increased STAT3-Ser727 phosphorylation in confluent, EGF-treated cells that was absent in cells that were either subconfluent or vehicle treated (Figure 8). Treatment of confluent, EGF-treated cells with the STAT3 specific inhibitor SPI completely abrogated EGF-stimulated VEGF production, indicating that STAT3, but not PI3K, is responsible for EGF-mediated VEGF-A production in confluent H292 cells (Figure 9). The dose of SPI used was verified as having no effect on cell viability (data not shown).

### 2.7. Gene Chip Analysis and Pathway Study

To confirm our finding that EGFR potentiates VEGF-A production in confluent cells and further elucidate differences in EGFR downstream signaling in confluent cells, we analyzed genome-wide differences in transcription by comparing relative transcription of individual genes between confluent and subconfluent cells treated with either saline or 100 ng/mL EGF. Relative to saline treatment, only 86 genes were differentially regulated in subconfluent cells treated with EGF while 231 genes were differentially regulated in confluent cells treated with EGF. Furthermore, 1267 genes were differentially regulated between EGF treated confluent *vs*. subconfluent cells (Table 1).

**Table 1:** Number of differentially regulated gene between subconfluent, confluent, and EGF (100 ng/mL) treated H292 cells after 24 h. Genes with expression changes >2-fold and *p* values < 0.05 are listed. Sample names are listed in the parentheses.

Data Set	Control Group (A)	Comparison Group (B)	Number of Differentially Regulated Genes (A *vs*. B)
1	Confluent + saline (NT50K 24 h)	Confluent + EGF (EGF50K 24 h)	231
2	Subconfluent + saline (NT10K 24 h)	Subconfluent + EGF (EGF10K 24 h)	86
3	Subconfluent + EGF (EGF10K 24 h)	Confluent + EGF (EGF50K 24 h)	1267

Upon further examination of these gene expression data, we discovered that VEGF-A mRNA was upregulated 2.8-fold in response to EGF treatment in confluent cells *vs*. EGF treated subconfluent cells. These results indicate a strong effect on gene modulation with EGF treatment in confluent, but not subconfluent cells and are in-line with our finding that VEGF-A protein secretion is enhanced in EGF-treated confluent cells.

To more precisely understand EGF-induced signaling in confluent cells we compared the list of differentially expressed genes between EGF-treated confluent and subconfluent cells (data set 3) to the list of differentially expressed genes between EGF-treated and untreated confluent cells (data set 1). The 112 genes shared between data sets 1 and 3 (Figure 9A) represent an enriched list of EGF responsive genes in confluent H292 cells. Using a heat map to compare expression patterns for these 112 genes between treatments, we found that the replicates (3×) for each treatments clustered very well with respect to gene expression patterns as evidence for data quality/reproducibility (Figure 9B). Gene expression patterns in response to EGF were substantially different between confluent and subconfluent samples, demonstrating that downstream effects of EGF signaling are distinct in confluent and subconfluent H292 cells.

**Figure 10:**
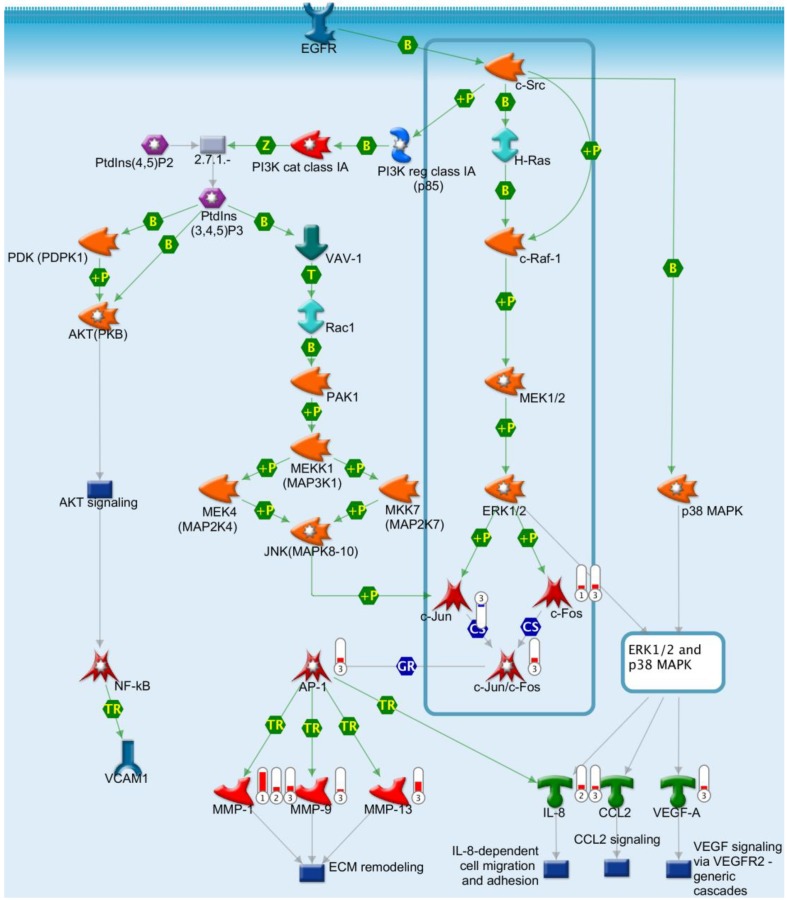
Pathway analysis. Genes differentially expressed between confluent and subconfluent conditions with EGF treatment were mapped in GeneGo database using MetaCore software. Label 1: data set 1; label 2: data set 2; label 3: data set 3 as listed in Table 1. The EGF signaling pathway was boxed in the graph. Each arrow indicates an interaction among molecules, green B for binding, green P for phosphorylation, blue CS for a complex subunit, green TR for transcription regulation.

To understand the effects of EGF on signal transduction in confluent *vs*. subconfluent H292 cells, the three sets of differentially expressed genes (Table 1) were mapped using MetaCore software for pathway analysis. The pathway analysis revealed angiogenesis in lung cancer as the top one hit for EGF induction in confluent but not subconfluent H292 cells. Three sets of data were mapped into the key pathway map. The pathway (Figure 10) indicates that EGFR potentially bound c-Src, and then subsequently activated H-Ras and c-Raf-1 pathways. In turn, c-Raf-1 phosphorylated mitogen-activated protein kinase MEK1/2, which then phosphorylated ERK1/2. The downstream gene c-Fos was upregulated and thus activated AP-1 (transcription factor) in EGF-treated confluent cells alone (data set 1 and 3). Consequently, in these EGF-treated confluent cells (data set 3), transcription factor AP-1 activated angiogenic genes, including metalloproteases *MMP-1*, *MMP-9* and *MMP-13* responsible for extracellular matrix (ECM) remodeling. In subconfluent H292 cells (data set 2), EGF was observed to induce *MMP-1* but no other ECM related genes, indicating a limited engagement of angiogenic functions as compared to confluent H292 cells. Therefore, EGFR induction supported ECM remodeling in both subconfluent and confluent cells, yet was synergistically enhanced with the combination of cell confluence and EGFR activation. Aside from pathways directly related to ECM remodeling, EGF induced growth factor transcription, including VEGF-A, in confluent cells (data set 3). As a whole, we conclude that confluent H292 cells are genetically predisposed to enhanced angiogenic activity with EGFR activation *vs.* subconfluent cells.

### 2.8. Potentiation of EGFR-Induced VEGF mRNA Production through Increased Cell Density Is Exclusive to H292 Cells

We extended our study to determine if the pathways presented here in H292 cells are broadly conserved across NSCLC derived from different patients and with different EGFR mutation status. Gene chip analysis of NSCLC lines H1993 (EGFR and cMet overexpression), A549 (KRAS mutant), and H1975 (L858R and T790M EGFR mutations) was used to determine whether these cell lines exhibited a similar potentiation of EGF-stimulated VEGF production in confluent cells. Our gene chip analysis did not reveal any statistically significant changes in VEGF mRNA in response to EGF when comparing sub-confluent *vs.* confluent cell populations (data not shown) in other cell lines, indicating that NSCLC expressing wild type EGFR, as in H292, are more likely to display the phenomena detailed in this study. While H292 cells are widely used as a good representation of the 80% of patients who exhibit wild type EGFR in NSCLC, tumor heterogeneity within wild type EGFR patients may limit the extent to which this phenomenon is observed in patient derived tumor samples. Future studies may utilize large repositories of patient-derived NSCLC lines to fully determine the extent to which this phenomenon extends clinically.

## 3. Experimental Section

### 3.1. Reagents & Cells

H292 cells were purchased from ATCC (Manassas, VA, USA). Human pulmonary microvascular endothelial cells (HPMEC) were purchased from Lonza and maintained in EBM2-MV (Lonza, Walkersville, MD, USA). H292 cells were maintained in RPMI media containing 10% FBS (Life Technologies, Carlsbad, CA, USA, 2 mM l-glutamine, 10 mM 4-(2-hydroxyethyl)-1-piperazineethanesulfonic acid (HEPES) (Life Technologies, Carlsbad, CA, USA), 1 mM sodium pyruvate, 4500 mg/L glucose, and 1500 mg/L sodium bicarbonate. HPMEC were maintained in EBM2-MV and starved in EBM2 (Lonza). LY-294002, PKH26 red and PKH67 green cell membrane labels were purchased from Sigma-Aldrich (St. Louis, MO, USA). Basement membrane matrix, growth factor reduced, phenol red free was purchased from BD Biosciences (San Jose, CA, USA). Anti-EGFR, anti-cMet and Hoechst stain were purchased from Cell Signaling Technology (Danvers, MA, USA) while anti-PIP2 antibody was purchased from Abcam (Cambridge, MA, USA). EGFR, cMet, and VEGF expression/phosphorylation kits were provided by Meso Scale Discovery (Rockville, MD, USA). SPI was purchased from EMD Millipore (Billerica, MA, USA). EGF, EGFR ECD, and phospho-protein blot were purchased from R&D Systems (Minneapolis, MN, USA).

### 3.2. Angiogenesis Assay

H292 cells were cultured to 90% confluence, harvested using Accutase (Life Technologies, Carlsbad, CA, USA), and stained green with PKH67 as per the manufacturers’ protocol. PKH67 stained cells were seeded onto transparent tissue culture treated 96-well plate as either a 2.5 μL spot containing 10,000 cells or as a 50 μL suspension containing 10,000 cells in RPMI. Surface tension was used to contain the 2.5 μL spot of H292 cells until both cell seeding suspensions had adhered to the plate (2 h) at which point the media volume was equalized to 50 μL and cells were chilled to 4 °C. Media was aspirated off chilled plates and cold phosphate-buffered saline (PBS), IgG, Cetuximab or EGFR ECD were added to H292 cells prior to overlay with 30 μL of pre-thawed growth factor reduced Matrigel matrix. Plates were then incubated at 37 °C until matrix solidified and complete growth media was added. After 16 h conditioning of matrix, media was removed and HMVEC-L stained with PKH26 and seeded as 40,000 cells in 100 μL of EBM2 media. After 12 h, plates were imaged using Operetta high content imaging system (PerkinElmer, Waltham, MA, USA).

### 3.3. Protein and Lipid Staining

H292 cells were seeded on poly-D coated glass bottom 96-well plates (PerkinElmer) as either 10,000 (subconfluent) or 50,000 (confluent) cells per well. After 48 h incubation at 37 °C, cells were fixed in 4% paraformaldehyde, permeabilized, and stained for EGFR, cMet, or PIP2 using primary fluorescently conjugated antibodies and Hoechst nuclear stain. Nuclei were stained with Hoechst. Antibody staining was validated by comparing all samples to fluorescently conjugated isotype controls. Images were acquired using Opera high content imaging system. Fluorescence intensities, membrane, cytosol, and nucleus identification/separation were calculated by Acapella software (PerkinElmer).

### 3.4. EGFR Phosphorylation

H292 cells were cultured to 90% confluence, harvested using Accutase, and seeded as either 10,000 or 50,000 cells/well of a 96 well plate in RPMI media. After 24 h, cells were preincubated with Cetuximab, PBS, or IgG for 30 min before being treated with increasing concentrations of EGF (0.1–100 nM) for 30 min. After EGF treatment, media was discarded, lysates were washed with phosphate buffered saline, and cells were lysed in 100 μL ice cold MSD lysis buffer supplemented with protease and phosphatase inhibitors and incubated on ice for 10 min and triturated until homogenous. Protein concentration of each well in the plate was determined via bicinchoninic acid (BCA) assay. Total and phosphor EGFR expression were then quantified using Phospho/Total EGFR whole cell lysate kit provided by Meso Scale Discovery using the manufacturers’ protocol.

### 3.5. VEGF Quantification

H292 cells were cultured to 90% confluence, harvested using Accutase, and seeded as either 10,000 or 50,000 cells/well of a 96 well plate in RPMI media. After 24 h, cells were preincubated with PBS, IgG, Cetuximab, WP1066, or LY294002 for 30 min before being treated with increasing concentrations of EGF (0.1–100 nM) for 30 min. After EGF treatment, media was aspirated off, lysates were washed with phosphate buffered saline, and cells were lysed in 100 μL ice cold MSD lysis buffer supplemented with protease and phosphatase inhibitors and incubated on ice for 10 min and triturated until homogenous. Protein concentration of each well in the plate was determined via BCA assay. VEGF in media was quantified using VEGF V-Plex kit provided by Meso Scale Discovery per the manufacturers’ protocol.

### 3.6. STAT3 Phosphorylation

H292 cells were grown in 100 mm plates with cell densities and media volumes calculated to reflect the conditions used in 96 well plates. Cells were treated with EGF for 30 min before removal of media, washing plate with PBS, and lysing cells. Protein concentration of lysate was quantified using BCA assay and 300 μg of protein from each treatment condition was used to determine phospho-protein expression using R&D Systems Proteome Profiler Human Phospho-Kinase Array kit.

### 3.7. Gene Expression

H292 cells were cultured to 90% confluence, harvested using Accutase, and seeded as either 10,000 or 50,000 cells/well of a 96 well plate in RPMI media. After 24 h, cells were treated with 100 ng/mL EGF in PBS or vehicle. RNA extraction was performed using RNeasy 96 well vacuum extraction kit (Qiagen, Hilden, Germany). Growth media was removed from cells and lysis was performed using cells and tissues lysing buffer (RLT) (Qiagen). Column extraction immediately followed and RNA was eluted in nuclease free water provided with the kit. RNA concentration and purity (A260/280 ratio) was determined using a Nanodrop spectrophotometer (Wilmington, DE, USA) and gene chip analysis was performed. Samples were processed at Janssen of Johnson and Johnson microarray facility in San Diego, CA, USA. Affymetrix GeneChip^®^ U133 HT human genome array (Santa Clara, CA, USA), which contains 50,000 probe sets (~33,000 genes and UniGene clusters) was used for the study. Data analysis was carried out using R package (Foundation for Statistical Computing, Vienna, Austria). ArrayQualityMetrics of R package was used for microarray data quality control. Data were normalized using Robust Multichip Average algorithm. Differentially expressed genes were obtained based on more than 2-fold changes and false discovery rate less than 0.05. Partek Genomics Suite (Partek Inc., Louis, MO, USA) was used for generating graphics. The pathway analyses were carried out using MetaCore software from Thomson Reuters (New York, NY, USA).

### 3.8. Statistics

All results are expressed as means ± standard error of the mean (SEM) Student’s *t*-test was used for simple comparisons between 2 data sets. One-way analysis of variance (ANOVA)with replication followed by a Bonferroni post-test was used to discern point differences in data sets with more than 2 groups. Statistical analyses and curve fits were performed using GraphPad Prism 5 software (GraphPad Software, La Jolla, CA, USA).

## 4. Conclusions

Our data indicate that within a monoclonal NSCLC cell line (H292), two distinctly different cell phenotypes can develop as a result of cell density. These individual phenotypes were characterized by their ability to potentiate endothelial cell angiogenesis in a paracrine fashion through co-culture models. Furthermore, we attribute this paracrine effect to differential responses to EGF ligand treatment and subsequent VEGF-A production. Cells seeded at confluence exhibited a 30% increase in EGFR phosphorylation in response to EGF treatment, followed by subsequent three-fold increases in VEGF-A mRNA and protein that were absent in subconfluent cells under identical treatment conditions. Subsequent inhibition of VEGF-A production via STAT3 inhibition but not PI3K inhibition indicated that STAT3 is a major contributor to EGF-stimulated VEGF-A production in confluent H292 cells. We confirmed our observations through mRNA gene expression analysis indicating upregulation of angiogenic signaling in confluent EGF-treated cells *vs.* subconfluent cells. Interestingly, these observations were not repeated in NSCLC cell lines H1993, A549, or H1975, indicating that this phenomenon may be exclusive to either wild type EGFR NSCLC lines or the H292 cell line alone. Nevertheless, these data identify a synergistic pathway through which EGFR stimulates VEGF-A production in confluent H292 cells as opposed to subconfluent cells. In summary, these results may shed light on the different mechanisms of resistance to anti-EGFR treatment in solid tumors experiencing high cell-cell contact *vs*. metastatic and circulating tumor cells experiencing minimal cell-cell contact. Future studies may evaluate the precise mechanism initiating separation of the subconfluent *vs*. confluent H292 cell phenotypes. Evaluation of the phenomena in various NSCLC cell lines as well as in patient samples is needed to confirm the relevance of these observations in clinical settings.
